# Does tinnitus amplify the effects of healthy eating patterns and physical activity on the sleep disturbance or sleep insufficiency, based on the case study of NHANES survey in the United States

**DOI:** 10.3389/fnut.2024.1427672

**Published:** 2024-08-29

**Authors:** Jia Chen, Wujun Zou, Hao Li, Yu Luo, Kaifu Lu, Xuelian Yi, Hong Li, Zhu Shi, Juan Meng

**Affiliations:** ^1^Department of Otorhinolaryngology, Head and Neck Surgery, The Second People’s Hospital of Chengdu, Chengdu, China; ^2^Department of Medical Cosmetology, The Second People’s Hospital of Chengdu, Chengdu, China; ^3^Department of Otorhinolaryngology, Head and Neck Surgery, The First People’s Hospital of Liangshan State, Xichang, Sichuan, China; ^4^Department of Otorhinolaryngology, Head and Neck Surgery, West China Hospital, West China School of Medicine, Sichuan University, Chengdu, China

**Keywords:** tinnitus, sleep disturbance, sleep insufficiency, dietary patterns, physical inactivity

## Abstract

**Objective:**

Exploring whether the presence of tinnitus amplifies the effects of an individual’s dietary patterns and physical activity on sleep disturbance or sleep insufficiency.

**Study design:**

This study extracted data from the five National Health and Nutrition Examination Surveys (NHANES) between 2009 and 2018, including individuals who had undergone complete questionnaires on tinnitus, dietary habits, physical activity, and sleep. Multivariate logistic regression, restricted cubic spline (RCS) and subgroup analyses were conducted to explore the associations of dietary habits, physical activity, and tinnitus with sleep disturbance and sleep insufficiency.

**Results:**

A total of 7,440 participants were enrolled in this study, of whom 1,795 participants were evaluated as sleep disturbance (24.13%), and 2,281 were sleep insufficiency (30.66%). With adjusting confounding factors of demographic and socioeconomic variables, among overall population, participants with tinnitus showed a significantly increased risk of sleep disturbance [adjusted odds ratio (aOR) = 2.08, 95% confidence interval (CI): 1.83–2.36), and sleep insufficiency (aOR = 1.31, 95% CI: 1.15–1.49). Poor dietary habits also increased the risk of sleep disturbance (aOR = 1.08, 95% CI: 1.04–1.12), as does lack of physical activity (aOR = 1.14, 95% CI: 1.03–1.27); but neither exposure factors significantly increased the risk of sleep insufficiency. The non-linear trend analyses of RCS found that the influence of exposure factors on sleep disturbance experiencing a steady or small decline trend after rising. In addition, the results of the subgroup analysis showed that in tinnitus patients, poor dietary habits and lack of physical activity both significantly increased the risk of sleep disturbance, and poor dietary habits also increased the risk of sleep insufficiency remarkable, but lack of physical activity did not. In healthy participants, poor dietary habits were only significantly associated the sleep disturbance, while lack of physical inactivity even had a protective effect against sleep insufficiency.

**Conclusion:**

Compared to the general population, tinnitus significantly amplified the effects of poor dietary patterns and physical inactivity on sleep disturbance and sleep insufficiency. For tinnitus patients, adjusting a healthy diet and increasing exercise could more effectively promote their sleep health.

## Introduction

Sleep disturbance is a group of conditions that disturb normal sleep patterns, commonly including insomnia, sleep-disordered breathing (SDB), circadian rhythm sleep-wake disorders (CRSD), parasomnias, and so forth (1); and sleep insufficiency is characterized as getting less sleep than necessary to maintain wakefulness during the daytime (2). Sleep disturbances and sleep insufficiency not only seriously affect people’s quality of life, but also have been confirmed to be closely associated with cardiovascular diseases and psychological diseases, such as hypertension, stroke, depression, social anxiety, etc. (3–7).

Over the past few decades, as modern lifestyles have changed and the pressure to study and work has increased, the effects of sleep disorders and insufficient sleep on people have gotten worse (8, 9). According to the report of the National Health and Nutrition Examination Survey (NHANES) conducted between 2005 and 2008, sleep insufficiency has become a prevalent condition, affecting approximately a third of the U.S. adult population (10). Following the COVID-19 pandemic, there has been an apparent increase in the prevalence of sleep disturbances and sleep insufficiency across a wide range of demographics, along with a corresponding rise in the disease burden, particularly among the older adults, mental health patients, physicians, nurses, students, etc. (11–15). A systematic review estimated that the global prevalence of post-COVID-19 sleep disturbances in adults could be 28.98% (25.73%–32.34%) (16).

Tinnitus is known as a widespread syndrome, which is the sensation of hearing noises such as buzzing or ringing in the absence of any external noise (17). Epidemiological surveys have revealed that the prevalence of tinnitus for adults in different regions is between 10% and 25% (18, 19). The most common variety of tinnitus is subjective, which generally caused by neurologic, otologic, metabolic, or psychogenic disorders; objective tinnitus is relatively uncommon and may indicate underlying vascular anomalies, neurologic illnesses, or dysfunction of the eustachian tube (20). Due to the constant, bothersome, and disturbing noises, tinnitus patients generally suffer the decreased life satisfaction, social interaction impairments, and occupational impairments (21). Notably, sleep disturbances is one of the most prevalent complications among tinnitus patients, a systematic review revealed that the pooled prevalence of sleep impairment in tinnitus patients may reach 53.5% with 95% confidence interval from 40.2% to 66.8% (22). Therefore, the healthy sleep of tinnitus patients is an essential research field that is well worth exploring.

Healthy lifestyle behaviors are essential for ensuring adequate sleep duration and a high-level of sleep quality (23), while the healthy diet and physical activity are two crucial dimensions evaluating healthy lifestyle behaviors. In recent years, some studies have found that the level of health diet is related to the sleep quality (24), and improved dietary patterns have a certain efficacy on improving the sleep quality (25, 26). In addition, physical activity has also been confirmed to have a strong association with healthy sleep (27), moderate-to-high intensity physical activity has a significant preventive effect on insufficient sleep and sleep disturbance (28–30).

Exploring the effects of tinnitus, dietary habits, and physical activity on sleep quality have been widely reported. However, whether the effects of dietary habits and physical activity on sleep quality differ between tinnitus patients and the general population is still unclear. This study aimed to assess the differences in the effects of dietary habits and physical activity on the sleep quality between these two populations, and provide a scientific basis for lifestyle behaviors adjustment to improve the sleep quality of tinnitus patients.

## Materials and methods

### Data source

The National Health and Nutrition Examination Survey (NHANES) database is a superior annual cross-sectional survey aiming to monitor the national’s nutrition and health in the United States. The Ethics Review Board of the National Center for Health Statistics (NCHS) authorized the study protocol and approved the use of the NHANES database,^1^ and all participants provided the written informed content. The multistage stratified probability sample based on selected counties, blocks, households, and individuals within households is utilized in the survey; and the probability weights are employed throughout the whole statistical analysis procedure in our work.

**FIGURE 1:**
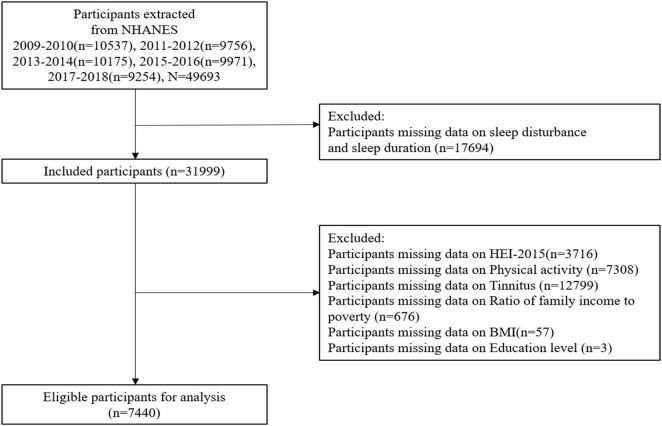
The flowchart of the participant inclusion and exclusion process.

For this study, we collected the data of dietary intake, physical activity monitor, tinnitus survey, and sleep quality during the five NHANES cycles between 2009 and 2018. Participants who were unable to complete dietary intake and physical activity interviews within 24 h were excluded, under the 16 years of age, as well as the absence information of self-reported sleep disorders questionnaire (SL-Q) and tinnitus. The unweighted response number of participants for all collected information were shown in the study’s flow chart, as shown in Figure 1.

### Outcome definitions

The self-reported prevalent outcomes that are associated with sleep disturbances were investigated using sleep problems and sleep duration. Sleep problems was determined based on the questionnaire item of “(Have you/Has SP) ever told a doctor or other health professional that (you have/s/he has) trouble sleeping?”. This item had two variants in different NHANES survey cycles, but with same evaluation content which not affected our determination of sleep problems. Participants who answered “yes” were considered to have a sleep disturbance, while those who answered “no” were thought to be free of sleep problems.

Furthermore, sleep duration was calculated using the item of “How much sleep do you get (hours)?”. According to the American Academy of Sleep Medicine (AASM) and the Sleep Research Society consensus recommend a minimum of 9 h for children 6–12 years of age, 8 h for adolescents, and at least 7 h for adults. Thus, participants was divided into adequate or insufficient sleep groups based on whether their sleep duration are less than 7 h (31). We also referenced other studies, further categorizing sleep duration as long (≥9 h), normal (7–9 h), and short (<7 h) (32).

### Exposures

The healthy dietary quality was assessed by the HEI-2015, which is a excellent tool to measures the alignment with the Dietary Guidelines for Americans (DGA) (33). For NHANES participants, their dietary recall interviews during the prior 24 h provided materials for the calculation of HEI-2015 score. The 13 dietary components of the HEI-2015 were composed with nine adequacy components (include total fruits, whole fruits, total vegetables, greens and beans, whole grains, dairy, total protein foods, seafood and plant proteins, and fatty acids) and four moderation components (include refined grains, sodium, added sugars, and saturated fats) (34). The higher score associated with higher intake level in the adequacy components and associated with lower intake level in the moderation components, and the total score of HEI-2015 ranged from 0 (no adherence) to 100 (best adherence) (35).

Metabolic equivalent (MET), which represented the ratio of the working metabolic rate to the resting metabolic rate, is a common indicator to express the relative energy metabolism level during various activities (36). NHANES provided the recommended MET values for different types of physical activity, including vigorous work-related activity (MET = 8), moderate work-related activity (MET = 4), walking or bicycling for transportation (MET = 4), vigorous leisure-time physical activity (MET = 8), and moderate leisure-time physical activity (MET = 4) (37). Besides, the physical activity questionnaire (PAQ) section provided the detailed records of various physical activities performed by NHANES participants during a typical week. Thus, in this study, the score of physical activity (PA) was calculated based on the MET value, activity type, weekly frequency, and duration. The score of PA was estimated according to the following formula:


PA⁢(MET-h/week)=MET×weekly⁢frequency×duration

where PA is a value greater than or equal to zero, and the higher value of PA means that participants with higher PA intensity. Participants in our study were divided into low intensity PA group (0–48 MET-h/week), and high intensity PA group (>48 MET-h/week) (38).

The presence of tinnitus was determined by recording the interview of the self-reported audiometry questionnaire (AUQ) (39). The questionnaire item of “In the past 12 months, (have you/has SP) been bothered by ringing, roaring, or buzzing in (your/his/her) ears or head that lasts for 5 minutes or more?” in the AUQ section provided the result of whether has tinnitus. Participants who answered “yes” were considered to have a tinnitus, while those who answered “no” were thought to be free of tinnitus.

### Covariates assessment

The covariates in this study included two parts: demographic characteristics and associated factors, and the associated factors were composed of healthy eating index (HEI), score of PAQ and the presence of tinnitus. Demographic characteristics were determined mainly through logical sorting and screening of previous studies. The variables included the individual information of age [1], sex [1], ratio of family income to poverty (which is used to measure the ratio of family income to the U.S. federal poverty line) (40), race (including non-Hispanic White, Mexican American, non-Hispanic Black, other Hispanic, or other race/multiple races) (40), education level (including high school graduate/GED or less, or more than high school), and body mass index (BMI), which include underweight, normal, overweight and obesity, according to the divided criteria of under 18.5, 18.5–24.9, 25–29.9, and over 30 kg/m^2^ (41).

### Statistical procedure

In the descriptive analysis, continuous variables were characterized by mean ± standard deviation (Mean ± SD), categorical variables were described as frequency combined with percentage (%), and Student’s *t*-test and Chi-square test were used to compare the difference for continuous and categorical variables between participants with sleep disturbance and normal participants respectively.

Furthermore, we utilized the multivariate logistic regression model to obtain the effect of HEI-2015, PA, and tinnitus on the sleep disturbance or insufficient sleep, using the odds ratio (OR) and 95% confidence interval (CI) to show the primary results. We set up five steps to gradually include the associated factors in our study based on the model constructed using the basic-demographic characteristics (including age, sex, race, ratio of family income to poverty, BMI, and education), which could more clearly exhibited the improvement effect of a specific associated factor on the whole model. Specifically, model 1 referred the basic-demographic variables model, model 2 included HEI-2015, model 3 included PA, model 4 included HEI-2015 and PA, and model 5 included HEI-2015, PA and tinnitus. Besides, the Net Reclassification Improvement (NRI) and Integrated Discrimination Improvement (IDI) indices were used to evaluate the improvement effect of model 2–5 comparing with model 1 (42).

Additionally, subgroup analysis focused on whether participants had tinnitus or not, and estimated the effects of HEI-2015 and PA on sleep disturbance and insufficient sleep in tinnitus patients and normal participants respectively, and conducted the comparison between groups to explore whether the effects of HEI-2015 and PA in tinnitus patients were significantly different from those in healthy participants. Lastly, the HEI-2015 and PA were transformed by restricted cubic spline (RCS) to characterized the non-linear trends of the association between them and sleep disturbance and sleep insufficiency respectively (43). Lastly, we conducted a sensitivity analysis for the three-category outcome of sleep duration (“long,” “normal,” and “short”) using multinomial logistic regression, with normal sleep duration (7–9 h) as the reference.

All analyses incorporated sample weights, stratification, and clustering of the complex sampling design to ensure nationally representative estimations. The whole process of analyses were performed using R version 4.3.2, and the significant level were set at a two-tailed α < 0.05.

## Results

Approximately, a weighted population of 356.3 million American residents was represented by 7,440 NHANES participants with valid data in our study. Among them, 3,531 participants were female (47.46%), while 3,909 were male (52.54%). A total of 993 were Mexican American (13.34%), 1,685 were non-Hispanic Black (22.65%), 2,972 were non-Hispanic White (39.95%), 715 were other Hispanic (9.61%), and 1,075 were other races (14.45%). The average ratio of family income to poverty was 2.53 with the SD of 1.65. In the distribution of BMI, 2,366 were normal (31.80%), 103 were underweight (1.38%), 2,365 were overweight (31.79%), and 2,606 were obesity (35.03%). The education degree of 3,224 participants (43.33%) was high school or below, and 4,216 participants were above high school (56.67%). The average score of HEI-2015 was 50.96 with the SD of 14.01. For the physical activity, the value of PA in 3,148 participants was more than 48 MET-h/week (42.31%), and 4,292 participants were less than 48 MET-h/week (57.69%). In addition, 1,132 (15.22%) participants were confirmed as tinnitus patients, 1,795 participants were sleep disturbance (24.13%), and 2,281 participants were sleep insufficiency (30.66%). Overall, the distributions of gender, age, BMI, physical activity and tinnitus were significantly different in the comparison of participants with or without sleep disturbance and sleep insufficiency, as details shown in Table 1.

**TABLE 1:** The description of demographic characteristics and associated factors for all participants divided by whether confirmed as sleep disturbance and sleep insufficiency.

Variables	Overall (*N* = 7,440)	Sleep disturbance			Sleep insufficiency		
		Sleep disturbance (*N* = 1,795)	General population (*N* = 5,645)	*p*-Value	Sleep insufficiency (*N* = 2,281)	General population (*N* = 5,159)	*p*-Value
Gender				< 0.001			.017
Female	3,531 (47.46%)	1,001 (55.77%)	2,530 (44.82%)		1,035 (45.38%)	2,496 (48.38%)	
Male	3,909 (52.54%)	794 (44.23%)	3,115 (55.18%)		1,246 (54.63%)	2,663 (51.62%)	
Age (mean ± SD, years)	44.34 ± 18.61	42.84 ± 18.67	49.05 ± 17.61	<0.001	45.31 ± 17.58	43.91 ± 19.03	<0.001
Race				0.181			0.894
Mexican American	993 (13.34%)	254 (14.15%)	739 (13.09%)		299 (13.11%)	694 (13.45%)	
Non-Hispanic Black	1,685 (22.65%)	378 (21.09%)	1,307 (23.15%)		518 (22.71%)	1,167 (22.62%)	
Non-Hispanic White	2,972 (39.95%)	710 (39.55%)	2,262 (40.07%)		899 (39.41%)	2,073 (40.18%)	
Other Hispanic	715 (9.61%)	172 (9.58%)	543 (9.62%)		224 (9.82%)	491 (9.52%)	
Other Races	1,075 (14.45%)	281 (15.66%)	794 (14.07%)		341 (14.95%)	734 (14.23%)	
Ratio of family income to poverty	2.53 ± 1.65	2.54 ± 1.65	2.53 ± 1.65	0.893	2.45 ± 1.64	2.57 ± 1.65	0.002
BMI				<0.001			<0.001
Normal	2,366 (31.80%)	460 (25.63%)	1,906 (33.76%)		611 (26.78%)	1,755 (34.02%)	
Underweight	103 (1.38%)	23 (1.28%)	80 (1.42%)		33 (1.45%)	70 (1.36%)	
Overweight	2,365 (31.79%)	531 (29.58%)	1,834 (32.49%)		757 (33.19%)	1,608 (31.17%)	
Obesity	2,606 (35.03%)	781 (43.51%)	1,825 (32.33%)		880 (38.58%)	1,726 (33.45%)	
Education				<0.001			0.178
High school graduate or below	3,224 (43.33%)	710 (39.55%)	2,514 (44.54%)		1,015 (44.50%)	2,209 (42.82%)	
Above high school	4,216 (56.67%)	1,085 (60.45%)	3,131 (55.46%)		1,266 (55.50%)	2,950 (57.18%)	
HEI-2015 score	50.96 ± 14.01	50.81 ± 14.00	51.01 ± 14.02	0.568	50.31 ± 13.63	51.25 ± 14.17	0.009
Physical activity (PA, MET-h/week)				<0.001			<0.001
High intensity level, PA > 48	3,148 (42.31%)	674 (37.55%)	2,474 (43.83%)		1,034 (45.33%)	2,114 (40.98%)	
Low intensity level, PA ≤ 48	4,292 (57.69%)	1,121 (62.45%)	3,171 (56.17%)		1,247 (54.67%)	3,045 (59.02%)	
Tinnitus				<0.001			<0.001
No	6,308 (84.78%)	1,327 (73.93%)	4,981 (88.24%)		1,882 (82.51%)	4,426 (85.79%)	
Yes	1,132 (15.22%)	468 (26.07%)	664 (11.76%)		399 (17.49%)	733 (14.21%)	

SD, standard deviation; PA, physical activity; BMI, body mass index; HEI-2015, 2015 version of healthy eating index; MET, metabolic equivalent.

For exploring the effects of healthy eating patterns and physical activity on the sleep quality, logistic regression analyses were conducted separately with setting outcomes as sleep disturbance and sleep insufficiency. Moreover, model 1–5 were utilized to progressively add the focused associated factors, reflecting the independent effect and stability of each associated factor, as details shown in Tables 2, 3, as well as in the Supplementary materials, Supplementary Tables 1, 2. Besides, the HEI-2015 score was preprocessed with the 100 minus HEI-2015 score divided by 10, the transformed HEI-2015 score ranged from 0 to 10, thus the higher score meant lower diet healthy.

**TABLE 2:** Logistic regression results regarding the impact of dietary patterns, physical activity, and tinnitus on sleep disturbance and sleep insufficiency.

Variables	Model adjusted all covariates for sleep disturbance			Model adjusted all covariates for sleep insufficiency		
	OR	95% CI (lower – upper)	*p*-Value	OR	95% CI (lower – upper)	*p*-Value
Gender			<0.001			<0.001
Female	Ref			Ref		
Male	0.69	0.62–0.76		1.26	1.14–1.40	
Age (years)	1.02	1.01–1.02	<0.001	1.00	0.99–1.01	0.200
Race			0.120			0.083
Mexican American	Ref			Ref		
Non-Hispanic Black	0.84	0.71–0.99		1.16	0.98–1.37	
Non-Hispanic White	0.90	0.77–1.05		1.09	0.93–1.27	
Other Hispanic	0.83	0.68–1.03		0.94	0.77–1.16	
Other Races	1.00	0.83–1.20		1.20	0.99–1.44	
Ratio of family income to poverty	0.97	0.94–1.00	0.051	0.92	0.89–0.95	<0.001
BMI			0.002			<0.001
Normal	Ref			Ref		
Underweight	1.43	0.91–2.20		1.20	0.74–1.88	
Overweight	1.07	0.94–1.22		1.51	1.33–1.71	
Obesity	1.25	1.10–1.42		1.37	1.20–1.55	
Education			<0.001			0.004
High school graduate or below	Ref			Ref		
Above high school	1.23	1.10–1.38		0.85	0.76–0.95	
HEI-2015 score	1.08	1.04–1.12	<0.001	1.02	0.98–1.06	0.400
Physical activity (PA, MET-h/week)			0.015			0.092
High intensity level, PA > 48	Ref			Ref		
Low intensity level, PA ≤ 48	1.14	1.03–1.27		0.92	0.83–1.01	
Tinnitus			<0.001			<0.001
No	Ref			Ref		
Yes	2.08	1.83–2.36		1.31	1.15–1.49	
Interaction of HEI-2015 and tinnitus	1.08	0.98–1.18	0.143	1.13	1.03–1.24	0.025
Interaction of PA and tinnitus	1.41	1.09–1.82	0.007	1.15	0.89–1.49	0.461

PA, physical activity; BMI, body mass index; HEI-2015, 2015 version of healthy eating index; MET, metabolic equivalent; Ref, reference; OR, odds ratios; CI, confidence interval.

**TABLE 3:** Performance improvement of logistic regression models with sequential addition of dietary patterns, physical activity, and tinnitus as exposure factors.

Outcomes	Models	NRI (95% CI)	IDI (95% CI)
Sleep disturbance	Basic model	Ref	
	Model added HEI-2015	0.0543 (0.0239–0.0836)	0.0016 (0.0005–0.0026)
	Model added PA	−0.0146 (−0.0243 to −0.0053)	0.0004 (−0.0002 to 0.0010)
	Model added HEI-2015 and PA	0.0456 (0.0103–0.0776)	0.002 (0.0007–0.0032)
	Model added all exposure factors	0.1885 (0.1443–0.231)	0.0212 (0.0174–0.0249)
Sleep insufficiency	Basic model	Ref	
	Model added HEI-2015	0.0002 (0–0.0006)	0.0002 (0–0.0005)
	Model added PA	0 (0–0)	0.0008 (0.0004–0.0013)
	Model added HEI-2015 and PA	0.0001 (−0.0070 to 0.0072)	0.0010 (0.0005–0.0016)
	Model added all exposure factors	0.0297 (0.0009–0.0566)	0.0023 (0.0011–0.0034)

NRI, Net Reclassification Improvement; IDI, Integrated Discrimination Improvement; PA, physical activity; HEI-2015, 2015 version of healthy eating index.

**FIGURE 2:**
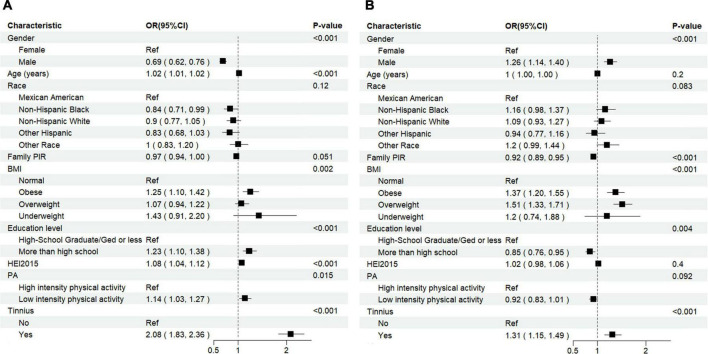
Forest plot of the logistic regression for the association between exposure factors with sleep disturbance **(A)** and sleep insufficiency **(B)**.

In terms of model results for sleep disturbance, the transformed HEI-2015 score was stably exhibited a significant association with sleep disturbance, with an adjusted OR value of 1.08 (95% CI: 1.04–1.12), the lower intensity level of physical activity was increased the risk of sleep disturbance, with the an adjusted OR of 1.14 (95% CI: 1.03–1.27), and the existence of tinnitus also was a considerable factor that increasing the risk of sleep disturbance (adjusted OR = 2.08, 95% CI: 1.83–2.36). In the view of sleep insufficiency, the transformed HEI-2015 score and the intensity of physical activity were both insignificant associated with the sleep insufficiency, but the adjusted risk of sleep insufficiency in tinnitus patients was still 1.31 times than that in the general population (95% CI: 1.15–1.49). From the model 2 to model 5 (Table 3), the effect sizes of HEI-2015 and PA on sleep disturbance and sleep insufficiency were stable, and the model 5 showed the most significant performance improvement compared to the basic model, with the NRI and IDI both reaching the maximum; and the forest plots for all associated factors are presented in Figure 2. The sensitivity analysis using multinomial logistic regression, with “normal” sleep duration as the reference, indicated that the association between tinnitus and “short” sleep duration remains consistent. The multinomial logistic regression results can be found in Supplementary Table 3. Furthermore, the interaction analyses in the fifth model for sleep disturbance and sleep insufficiency both showed that healthy dietary patterns and physical activity had significant interaction effect with tinnitus. Therefore, subgroup analyses were further performed according to the presence or absence of tinnitus. The forest plots for subgroup analyses are presented in Supplementary Figures 1, 2.

The results of subgroup analyses are detailed in Table 4. Comparing the effect value of transformed HEI-2015 score and physical activity value on sleep disturbance in tinnitus patients and general population, it could be found that the effect size of HEI-2015 and PA in tinnitus patients are both higher than that in general people (OR of HEI-2015 in tinnitus: 1.11 vs. in general people: 1.06; OR of lower intensity PA level in tinnitus: 1.58 vs. in general people: 1.05). In the subgroup comparison of sleep insufficiency, only HEI-2015 score expressed a significant effect on insufficient sleep in tinnitus patients (OR = 1.13, 95% CI: 1.04–1.24), and with insignificant effect in the general population, for another, the low intensity of PA even reduced the risk of sleep insufficiency in general people with OR value of 0.89 (95% CI: 0.80–1.00), and showed insignificant effect in tinnitus patients. The forest plots for subgroup analyses are presented in Supplementary Figures 1, 2.

**TABLE 4:** The effect size of dietary patterns and physical activity on the sleep disturbance and sleep insufficiency in tinnitus patients compared with the general population.

Variables	Sleep disturbance						Sleep insufficiency					
	Tinnitus patients			General population			Tinnitus patients			General population		
	OR	95% CI	*p*-Value	OR	95% CI	*p*-Value	OR	95% CI	*p*-Value	OR	95% CI	*p*-Value
Gender			<0.001			<0.001			0.927			<0.001
Female	Ref			Ref			Ref			Ref		
Male	0.56	0.44–0.70		0.73	0.65–0.82		0.99	0.77–1.26		1.34	1.20–1.50	
Age (years)	1.01	1.00–1.02	0.018	1.02	1.01–1.02	<0.001	1.00	0.99–1.01	0.910	0.99	0.99–1.00	0.212
Race			<0.001			0.005			0.173			0.043
Mexican American	Ref			Ref			Ref			Ref		
Non-Hispanic Black	0.71	0.49–1.03		0.87	0.72–1.06		0.93	0.64–1.37		1.22	1.01–1.48	
Non-Hispanic White	0.58	0.41–0.82		0.99	0.84–1.19		0.70	0.49–1.00		1.21	1.01–1.44	
Other Hispanic	1.28	0.82–1.98		0.75	0.59–0.96		0.85	0.53–1.33		0.99	0.78–1.25	
Other Races	0.62	0.41–0.94		1.12	0.91–1.38		1.01	0.66–1.53		1.26	1.02–1.55	
Ratio of family income to poverty	0.85	0.78–0.91	<0.001	0.99	0.96–1.04	0.932	0.87	0.80–0.94	<0.001	0.93	0.90–0.97	<0.001
BMI			0.780			0.002			0.411			<0.001
Normal	Ref			Ref			Ref			Ref		
Underweight	1.92	0.55–7.54		1.43	0.87–2.28		1.05	0.26–3.59		1.27	0.75–2.05	
Overweight	1.02	0.75–1.39		1.05	0.91–1.21		1.20	0.87–1.65		1.56	1.36–1.79	
Obesity	1.05	0.78–1.41		1.28	1.11–1.48		0.95	0.70–1.29		1.46	1.27–1.69	
Education			0.004			0.003			0.534			0.007
High school graduate or below	Ref			Ref			Ref			Ref		
Above high school	1.46	1.13–1.89		1.22	1.07–1.39		0.92	0.71–1.19		0.85	0.75–0.96	
HEI-2015 score	1.11	1.02–1.21	0.021	1.06	1.02–1.11	0.004	1.13	1.04–1.24	0.007	0.99	0.95–1.03	0.740
Physical activity (PA, MET-h/week)			<0.001			0.394			0.835			0.049
High intensity level, PA > 48	Ref			Ref			Ref			Ref		
Low intensity level, PA ≤ 48	1.58	1.24–2.02		1.05	0.94–1.18		1.03	0.80–1.32		0.89	0.80–1.00	

PA, physical activity; BMI, body mass index; HEI-2015, 2015 version of healthy eating index; MET, metabolic equivalent; Ref, reference; OR, odds ratio; CI, confidence interval.

**FIGURE 3:**
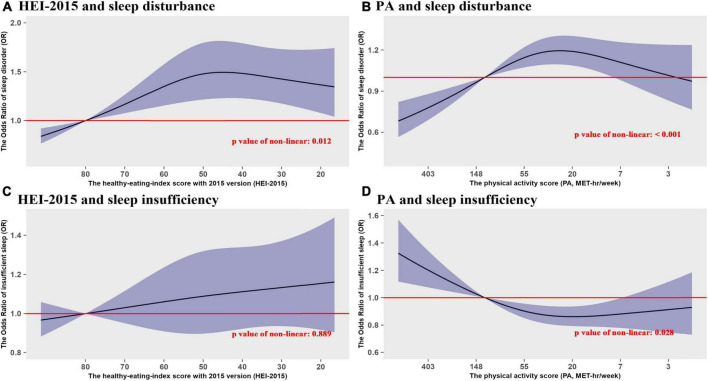
The non-linear trend analyses of the effects of dietary patterns and physical activity on sleep disturbance and sleep insufficiency. **(A)** HEI-2015 and sleep disturbance. **(B)** Physical activity (PA) and sleep disturbance. **(C)** PA and sleep insufficiency. **(D)** PA and sleep insufficiency.

Lastly, considering that the data distribution of original PA value is discrete, a negative logarithm transformation was performed on it, with a higher PA value meant lower intensity of physical activity, and based on this, the transformed HEI-2015 score and PA value were used to analyze the non-linear trends for sleep disorder and insufficient sleep. When the scores of HEI-2015 and PA declined, the effects of HEI-2015 and PA on sleep disturbance both exhibited significant non-linear trends, with a trend of rising first and than stabilizing or decreasing slightly. For the sleep insufficiency, the effect of HEI-2015 showed a approximately linear trend, while the PA showed a significant non-linear trend of first decreasing and then stabilizing. The non-linear trends for the full population are exhibited in Figure 3. For the subgroup analyses of non-linear trends in tinnitus patients and general people, the non-linear trends of HEI-2015 and PA both showed a smoother upward trend in tinnitus patients comparing with the general population, as details shown in Supplementary Figures 3, 4.

## Discussion

This study was the first time to explore the difference effects of the healthy eating patterns and physical activity on the sleep quality between tinnitus patients and normal participants. For the sleep disturbance, the lower values of HEI-2015 and PA were associated with a higher risk of sleep disturbance; and tinnitus also was a significant influencing factor that cannot be ignored, causing the effect of eating patterns and physical activity in patients with tinnitus on the sleep disturbance were considerably stronger than general population, especially for the physical activity. For the sleep insufficiency, the HEI-2015 and PA did not show a significant association with sleep insufficiency in the overall population, but the tinnitus was significantly associated with higher risk of sleep insufficiency. Moreover, the HEI-2015 in tinnitus patients showed a significant effect on sleep insufficiency, which was different from that in the general population.

In addition, the RCS analyses found a considerable non-linear trend in the association between HEI-2015 and sleep disturbance, but with insignificant non-linear trend in the HEI-sleep-insufficiency association. Although the non-linear trends were both significant in the association of PA value with sleep disturbance and sleep insufficiency, the effect size changed in opposite direction as PA decreased, and these associations also exhibited considerable differences between tinnitus patients and general population. In addition, obesity was also found significantly associated with sleep disturbance and sleep insufficiency, especially in the general population.

Numerous studies have demonstrated that inflammatory factors such as interleukin 1 beta (IL-1β), IL-6, IL-8, tumor necrosis factor alpha (TNF-α), C-reactive protein (CRP), and monocyte chemotactic protein-1 (MCP-1) are key molecules extensively involved in the body’s inflammatory responses and immune regulation (44–46). Inflammation plays a pivotal role in the association of dietary habits, PA and tinnitus with the sleep disturbance and sleep insufficiency by modulating various immune factors such as cytokines, chemokines, and reactive oxygen species (ROS) (47, 48). These inflammatory mediators can exacerbate tinnitus symptoms by promoting neuroinflammation and oxidative stress within the auditory pathway. Elevated levels of cytokines such as IL-1β, IL-6, and TNF-α have been shown to disrupt sleep architecture by reducing slow-wave sleep and increasing sleep fragmentation (49, 50). Inadequate sleep further exacerbates the inflammatory response, creating a vicious cycle that perpetuates poor health outcomes (51). This interconnected mechanism underscores the importance of maintaining healthy dietary and physical activity habits to mitigate the effects of tinnitus and improve sleep quality and duration.

For instance, poor dietary habits, characterized by high intake of saturated fats and sugars, can induce systemic inflammation through increased production of pro-inflammatory cytokines like IL-8, IL-6, TNF-α, CRP, and ROS (52, 53), while these inflammatory responses negatively impacts sleep quality and duration. In addition, obesity often results from poor dietary habits, which can further exacerbate inflammation and oxidative stress, both of which are known to disrupt sleep (54). Some studies also found that elevated levels of pro-inflammatory cytokines, such as IL-1β, IL-6, and TNF-α, associated with obesity can impair sleep architecture and promote sleep disturbances (55).

In our study’s findings, the adverse effect of lower HEI-2015 scores on the risk of sleep disturbance was stronger in tinnitus patients than that in the general population, which means that unhealthy eating patterns could bring higher health risks and more serious health damage for tinnitus patients. Besides, lower HEI-2015 scores were also associated with significantly shorter sleep duration in tinnitus patients, but not in the general population, which also means that unhealthy eating patterns could also significantly shorten the sleep duration and reduce the quality of life for tinnitus patients. We thought that the existence of tinnitus may potentially lead to the imbalance of patient’s emotion regulation, aggravate the patient’s psychological pressure and emotional anxiety, these symptoms could induce the changes of pro-inflammatory cytokines in the endocrine system and hormone levels, and further exacerbate the impact of poor diet on sleep quality (55, 56).

Similarly, chronically low levels of physical activity and sedentary behavior can lead to heightened inflammation by rising anti-inflammatory cytokines like IL-6, IL-10, and TNF-α and increasing ROS production (57, 58). Combined with our findings, lower intensity PA was significantly associated with the higher risk of sleep disturbance, but the association between PA value and sleep duration was insignificant. In addition, the adverse effect of lower intensity level of PA on sleep disturbance was mainly concentrated in tinnitus patients rather than general population. These findings suggested that in the general population, lower levels of physical activity may not significantly interfered with sleep quality, but may extend sleep duration, but in tinnitus patients, lower levels of physical activity may significantly increased the risk of sleep disorders. This finding suggested that for tinnitus patients, increasing daily physical activity is helpful to reduce the risk of sleep disorders.

Overall, the interaction between poor dietary habits, physical inactivity and tinnitus in association to sleep disturbance underscores the importance of optimizing diet pattern and strengthening regular exercise in this population (59, 60). Poor dietary habits, obesity and physical inactivity not only acts as an independent risk factor but also exacerbates the negative impact of sleep disturbance in tinnitus patients, thereby magnifying the challenges faced by tinnitus patients in managing sleep-related issues (61). Moreover, two randomized controlled trials (RCTs) conducted by Özbey-Yücel and Uçar (61) show that dietary and physical activity interventions, whether conducted alone or in combination, can alleviate tinnitus symptoms (62, 63). Therefore, future research comparing the inflammatory processes of poor dietary habits and physical inactivity between tinnitus patients and the general population is essential and can provide valuable insights into the underlying mechanisms.

### Limitations

There are several limitations to this study. Firstly, the diet assessment might involve measurement errors and inaccuracies. Secondly, physical activity measures do not reflect the regularity of an individual’s physical activity. Moreover, there are many forms of physical activity, and the measurement of its degree and frequency is often difficult to achieve an accurate and uniform standard. The MET used in our study can only reflect the average daily exercise intensity of an individual, but cannot reflect whether the type of exercise is acute or regular (64). In previous literature reviews, regular exercise and moderate physical activity have a good effect on improving sleep quality, but have a limited effect on overall sleep duration (65). Lastly, bias is inevitable in cross-sectional studies.

## Conclusion

Compared to the general population, the presence of tinnitus significantly amplified the effects of poor dietary patterns and physical inactivity on sleep disturbance and sleep insufficiency. For tinnitus patients, adjusting a healthy diet and increasing exercise could more effectively promote their sleep health. Future research should explore the underlying mechanisms between these interventions and sleep quality improvements in this group.

## Data Availability

The data used in this study are publicly available as part of the National Health and Nutrition Examination Survey, which is distributed and sponsored by the Centers for Disease Control and Prevention (https://www.cdc.gov/nchs/nhanes/index.htm).

## Footnotes

1
https://www.cdc.gov/nchs/nhanes/
