# Supplementary material for: Does tinnitus amplify the effects of healthy eating patterns and physical activity on the sleep disturbance or sleep insufficiency, based on the case study of NHANES survey in the United States
Source: Front Nutr. 2024 Aug 29;11:1427672. doi: 10.3389/fnut.2024.1427672 (PMC11390667; doi:10.3389/fnut.2024.1427672)
Supplement: Supplementary file 1 [file Data_Sheet_1.pdf]

Supplementary materials

The supplementary materials section of this study includes forest plots for separate subgroup analyses of the outcome variables of insufficient sleep and sleep disturbance, according to the presence or absence of tinnitus, and shows the results of nonlinear trend analyses in all subgroup analyses. Additionally, the supplementary materials contain the logistic regression results for Models 1-4 of sleep disturbance and insufficient sleep, as well as the multinomial logistic regression results for insufficient sleep.

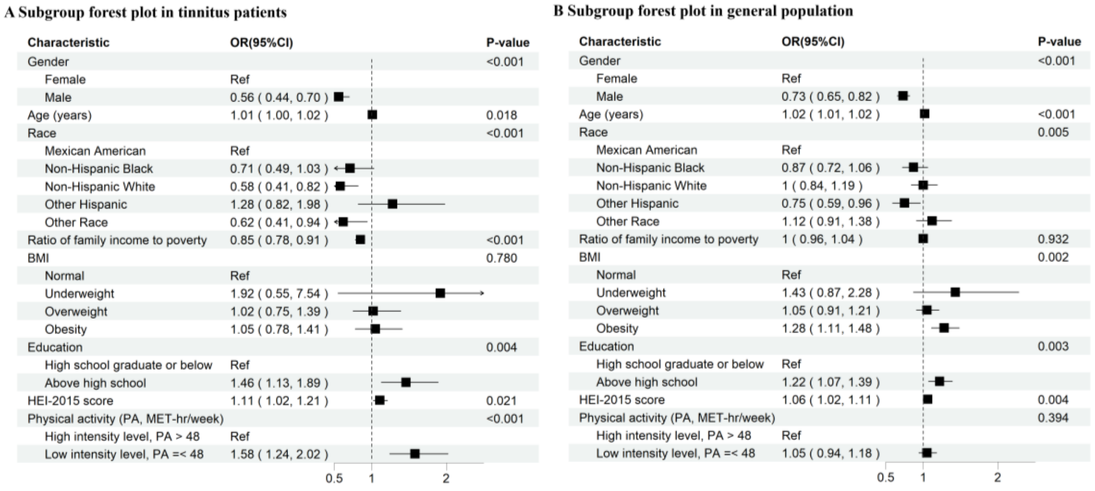

Figure Sup 1. Subgroup analysis forest plot of the association between associated factors with sleep disturbance.

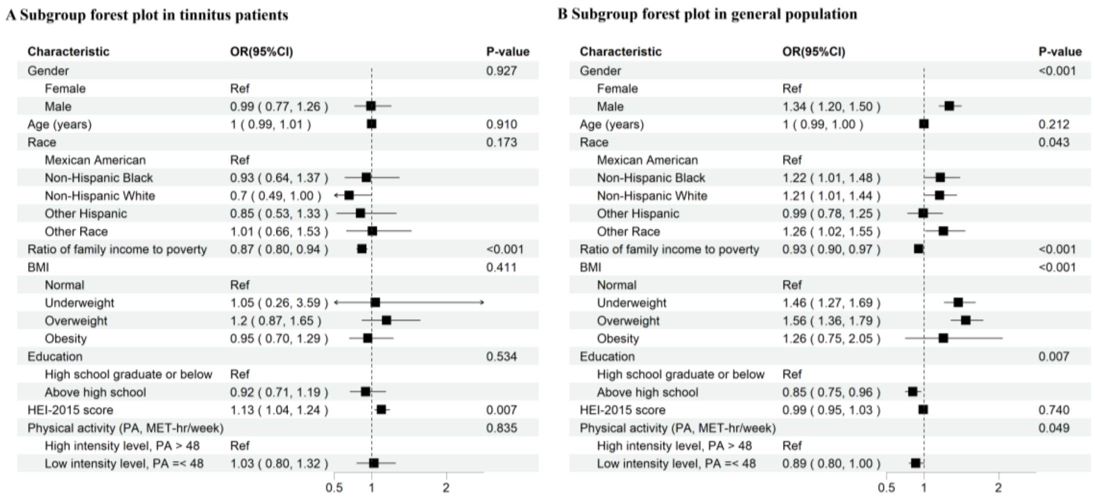

Figure Sup 2. Subgroup analysis forest plot of the association between associated factors with sleep insufficiency.

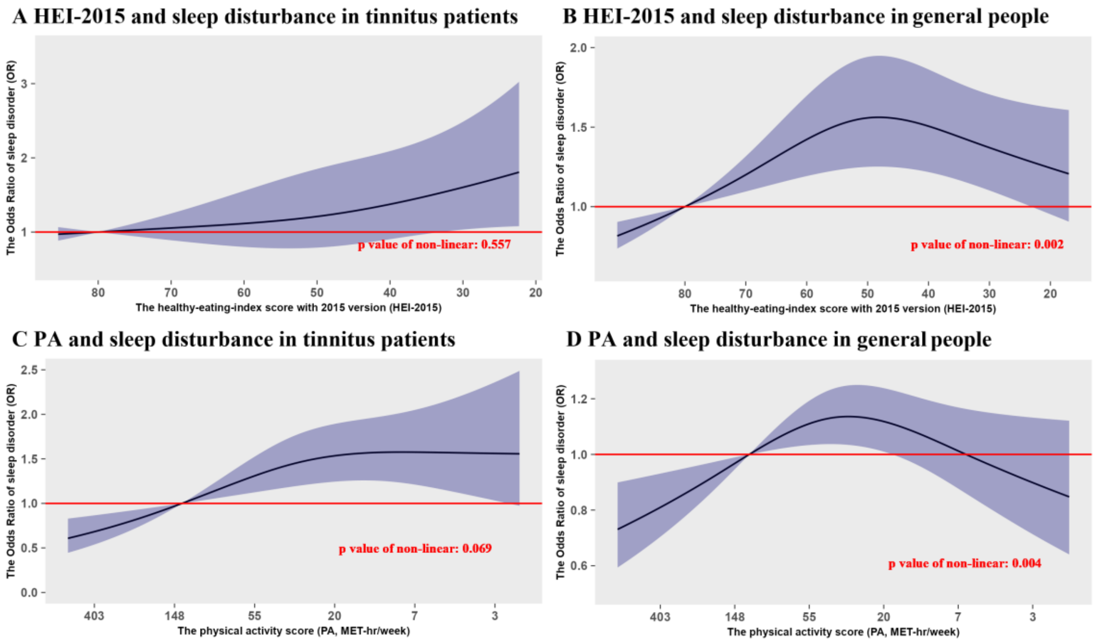

Figure Sup 3. The non-linear trend subgroup analyses of the effects of healthy dietary patterns and physical activity on sleep disturbance.

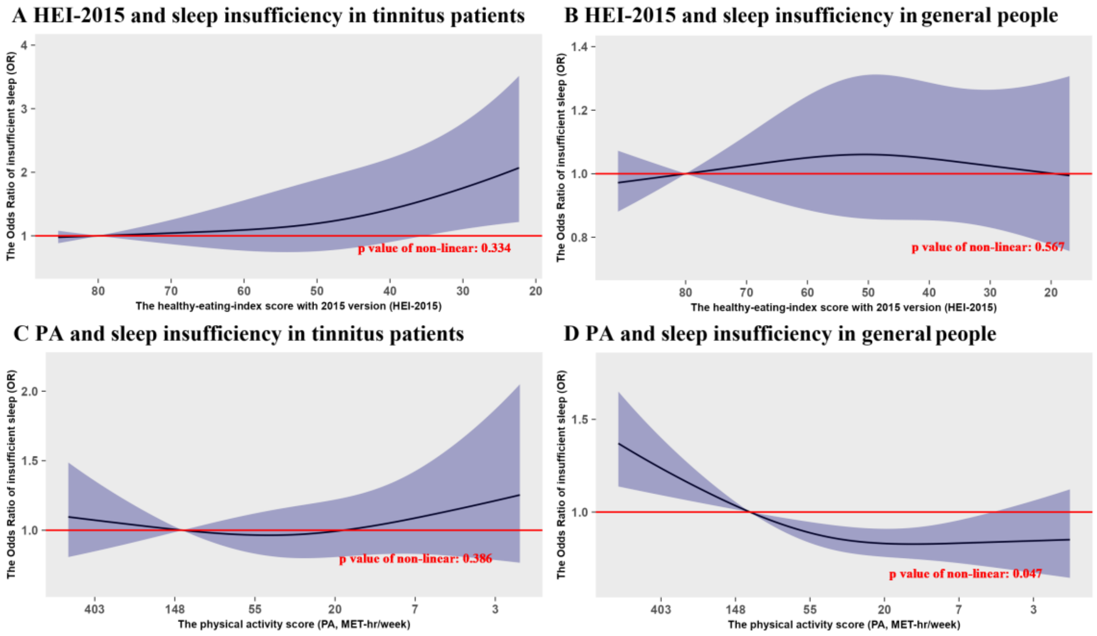

Figure Sup 4. The non-linear trend subgroup analyses of the effects of healthy dietary patterns and physical activity on sleep insufficiency.

Table Sup1. The results of logistic regression for the association between associated factors on sleep disturbance

Variables	Basic model			Model added HEI-2015			Model added PA			Model added HEI-2015 and PA		
	**OR**	**95% CI**	***p* value**	**OR**	**95% CI**	***p* value**	**OR**	**95% CI**	***p* value**	**OR**	**95% CI**	***p* value**
Gender			< .001			< .001			< .001			< .001
Female	ref			ref			ref			ref		
Male	0.72	0.65 - 0.79		0.7	0.64 - 0.78		0.73	0.66 - 0.81		0.72	0.65 - 0.79	
Age (years)	1.02	1.02 - 1.02	< .001	1.02	1.02 - 1.02	< .001	1.02	1.02 - 1.02	< .001	1.02	1.02 - 1.02	< .001
Race			0.089			0.088			0.082			0.082
Mexican American	ref			ref			ref			ref		
Non Hispanic Black	0.83	0.70 - 0.98		0.83	0.70 - 0.98		0.82	0.70 - 0.97		0.82	0.70 - 0.97	
Non Hispanic White	0.87	0.75 - 1.02		0.87	0.75 - 1.02		0.87	0.75 - 1.02		0.87	0.75 - 1.02	
Other Hispanic	0.83	0.68 - 1.02		0.84	0.69 - 1.03		0.83	0.67 - 1.01		0.84	0.68 - 1.02	
Other Races	0.98	0.82 - 1.18		0.99	0.82 - 1.19		0.98	0.81 - 1.17		0.98	0.82 - 1.18	
Ratio of family income to poverty	0.96	0.93 - 0.99	0.01	0.96	0.93 - 1.00	0.028	0.96	0.93 - 0.99	0.007	0.96	0.93 - 0.99	0.02
BMI			< .001			< .001			< .001			< .001
Normal	ref			ref			ref			ref		
Underweight	1.52	0.97 - 2.33		1.47	0.94 - 2.25		1.49	0.96 - 2.29		1.44	0.92 - 2.21	
Overweight	1.09	0.96 - 1.24		1.07	0.94 - 1.22		1.08	0.95 - 1.23		1.07	0.94 - 1.22	
Obesity	1.35	1.19 - 1.53		1.31	1.15 - 1.48		1.34	1.19 - 1.52		1.29	1.14 - 1.47	
Education			0.005			0.001			0.007			0.002
High school graduate or below	ref			ref			ref			ref		
Above high school	1.17	1.05 - 1.32		1.21	1.08 - 1.35		1.17	1.04 - 1.31		1.20	1.07 - 1.35	
HEI-2015 score				1.08	1.04 - 1.12	< .001				1.08	1.04 - 1.12	< .001
Physical activity (PA, MET-hr / week)									0.027			0.021
High intensity level, PA > 48							ref			ref		

Low intensity level, PA <= 48							1.13	1.01 - 1.25		1.13	1.02 - 1.26	

Notes: SD, standard deviation; PA, physical activity; BMI, body mass index; HEI-2015, 2015 version of healthy eating index; MET, metabolic equivalent.

Table Sup2. The results of logistic regression for the association between associated factors on sleep insufficiency

Variables	Basic model			Model added HEI-2015			Model added PA			Model added HEI-2015 and PA		
	**OR**	**95% CI**	***p* value**	**OR**	**95% CI**	***p* value**	**OR**	**95% CI**	***p* value**	**OR**	**95% CI**	***p* value**
Gender			< .001			< .001			< .001			< .001
Female	ref			ref			ref			ref		
Male	1.30	1.18 - 1.44		1.29	1.17 - 1.43		1.28	1.16 - 1.42		1.28	1.15 - 1.41	
Age (years)	1.00	0.99 - 1.01	0.300	1.00	0.99 - 1.01	0.500	1.00	0.99 - 1.01	0.500	1.00	0.99 - 1.01	0.600
Race			0.110			0.110			0.100			0.100
Mexican American	ref			ref			ref			ref		
Non Hispanic Black	1.14	0.96 - 1.35		1.14	0.97 - 1.35		1.15	0.97 - 1.36		1.15	0.97 - 1.36	
Non Hispanic White	1.07	0.92 - 1.26		1.07	0.92 - 1.26		1.08	0.92 - 1.26		1.08	0.92 - 1.26	
Other Hispanic	0.94	0.76 - 1.15		0.94	0.76 - 1.15		0.94	0.76 - 1.16		0.94	0.77 - 1.16	
Other Races	1.18	0.98 - 1.42		1.18	0.98 - 1.43		1.19	0.99 - 1.43		1.19	0.99 - 1.43	
Ratio of family income to poverty	0.92	0.89 - 0.95	< .001	0.92	0.89 - 0.95	< .001	0.92	0.89 - 0.95	< .001	0.92	0.89 - 0.95	< .001
BMI			< .001			< .001			< .001			< .001
Normal	ref			ref			ref			ref		
Underweight	1.20	0.74 - 1.87		1.19	0.73 - 1.86		1.22	0.75 - 1.90		1.20	0.75 - 1.89	
Overweight	1.50	1.33 - 1.71		1.50	1.32 - 1.70		1.51	1.33 - 1.71		1.51	1.33 - 1.71	
Obesity	1.39	1.22 - 1.57		1.37	1.21 - 1.56		1.39	1.23 - 1.58		1.38	1.22 - 1.57	
Education			0.001			0.002			0.002			0.003
High school graduate or below	ref			ref			ref			ref		
Above high school	0.84	0.75 - 0.93		0.84	0.76 - 0.94		0.84	0.75 - 0.94		0.85	0.76 - 0.94	
HEI-2015 score				1.02	0.98 - 1.06	0.300				1.02	0.98 - 1.06	0.300

Physical activity (PA, MET-hr / week)			0.081			0.086
High intensity level, PA > 48	ref			ref		
Low intensity level, PA <=48	0.91	0.82 - 1.01		0.91	0.82 - 1.01	

Notes: SD, standard deviation; PA, physical activity; BMI, body mass index; HEI-2015, 2015 version of healthy eating index; MET, metabolic equivalent.

Table Sup3. The results of multinomial logistic regression

Classifications	Variables	OR	95% CI lower	95%CI upper	*p* value
	Gender				< .001
	Female	ref			
	Male	0.720	0.628	0.826	
	Age (years)	1.000	0.996	1.004	0.996
	Race				
	Mexican American	ref			
	Non Hispanic Black	1.174	0.942	1.462	0.153
	Non Hispanic White	0.864	0.702	1.063	0.168
Sleep excessiveness	Other Hispanic	0.803	0.608	1.060	0.121
	Other Races	1.118	0.877	1.425	0.369
	Ratio of family income to poverty	0.831	0.796	0.868	< .001
	BMI				
	Normal	ref			
	Underweight	1.000	0.848	1.179	0.999
	Overweight	1.040	0.880	1.230	0.644
	Obesity	2.773	1.716	4.481	0.000
	Education				< .001
	High school graduate or below	ref			

	Above high school	0.711	0.616	0.822	
	HEI-2015 score	1.118	1.063	1.175	< .001
	Physical activity (PA, MET-hr / week)				0.534
	High intensity level, PA > 48	ref			
	Low intensity level, PA <= 48	0.957	0.834	1.099	
	**Tinnitus**				**0.667**
	**No**	**ref**			
	**Yes**	**0.959**	**0.795**	**1.158**	
	Gender				< .001
	Female				
	Male	1.187	1.068	1.319	
	Age (years)	0.998	0.995	1.001	0.248
	Race				
	Mexican American				
	Non Hispanic Black	1.201	1.008	1.431	0.041
	Non Hispanic White	1.053	0.895	1.239	0.532
Sleep insufficiency	Other Hispanic	0.918	0.741	1.137	0.434
	Other Races	1.227	1.012	1.489	0.037
	Ratio of family income to poverty	0.890	0.861	0.920	< .001
	BMI				
	Normal				
	Underweight	1.396	1.223	1.593	< .001
	Overweight	1.530	1.342	1.745	< .001
	Obesity	1.679	1.015	2.776	0.043
	Education				< .001

High school graduate or below				
Above high school	0.796	0.710	0.893	
HEI-2015 score	1.039	1.000	1.079	0.052
Physical activity (PA, MET-hr / week)				0.051
High intensity level, PA > 48				
Low intensity level, PA <= 48	0.899	0.808	1.000	
**Tinnitus**				**< .001**
**No**				
**Yes**	**1.281**	**1.118**	**1.468**	
